# Ups and Downs in 75 Years of Electrocochleography

**DOI:** 10.3389/fnsys.2017.00002

**Published:** 2017-01-24

**Authors:** Jos J. Eggermont

**Affiliations:** ^1^Department of Psychology, University of CalgaryCalgary, AB, Canada; ^2^Department of Physiology and Pharmacology, University of CalgaryCalgary, AB, Canada

**Keywords:** auditory nerve, summating potential, compound action potential, cochlear microphonic, Ménière’s disease, vestibular schwannoma, auditory neuropathy, cochlear implants

## Abstract

Before 1964, electrocochleography (ECochG) was a surgical procedure carried out in the operating theatre. Currently, the newest application is also an intra-operative one, often carried out in conjunction with cochlear implant surgery. Starting in 1967, the recording methods became either minimal- or not-invasive, i.e., trans-tympanic (TT) or extra tympanic (ET), and included extensive studies of the arguments pro and con. I will review several valuable applications of ECochG, from a historical point of view, but covering all 75 years if applicable. The main topics will be: (1) comparing human and animal cochlear electrophysiology; (2) the use in objective audiometry involving tone pip stimulation—currently mostly pre cochlear implantation but otherwise replaced by auditory brainstem response (ABR) recordings; (3) attempts to diagnose Ménière’s disease and the role of the summating potential (SP); (4) early use in diagnosing vestibular schwannomas—now taken over by ABR screening and MRI confirmation; (5) relating human electrophysiology to the effects of genes as in auditory neuropathy; and (6) intracochlear recording using the cochlear implant electrodes. The last two applications are the most recently added ones. The “historical aspects” of this review article will highlight the founding years prior to 1980 when relevant. A survey of articles on Pubmed shows several ups and downs in the clinical interest as reflected in the publication counts over the last 75 years.

## Introduction

**Figure 1:**
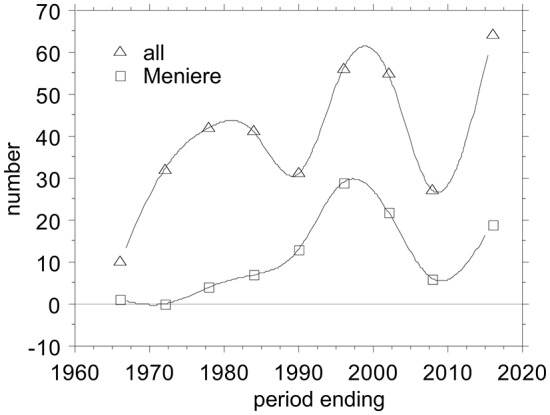
**Ups and Downs in the number of publications on Electrocochleography (ECochG), overall and applied to Ménière’s disease.** I tabulated all ECochG articles between 1941 and present that I could find in PubMed and added those from a 1976 conference proceeding (Ruben et al., 1976), amounting to 358 publications covering 75 years. I grouped them in periods of 6 years, because that bracketed the early minimum-intervention recording period from 1967–1972. The first 25 years before that I grouped together. The last period ending July 2016 covers 6.5 years.

Electrocochleography (ECochG) is a technique for recording sound-evoked cochlear and auditory nerve population responses from the round window, the cochlear wall (promontory), eardrum and external ear canal. One observes (Figure 1) that there are several ups and downs in the number of publications across the years, potentially reflecting the waxing and waning interest for ECochG as a diagnostic tool. Overall, there is a trend for a slow increase in the output.

### Early Surgical Recordings

The first indications of the feasibility of recording cochlear potentials came from Fromm et al. (1935); the responses obtained in two humans with perforated eardrums were small and no cathode ray display could be obtained. Improved recording and amplification techniques gave better cochlear microphonics (CM) recordings (Perlman and Case, 1941; Lempert et al., 1947, 1950). Perlman and Case (1941) placed an electrode on the cochlea, first in monkeys and later in human ears. They found that CM could be obtained regularly in humans with a nearly normal audiogram. The potentials could be clearly detected in a loudspeaker or headphones. I consider this the start of ECochG, albeit that only later Lempert et al. (1947) coined the term “cochleogram”. They carried out recordings in 11 human ears in the course of surgical interventions for otosclerosis, tinnitus or Ménières’ disease. They could record responses from the round window in six ears but not from the promontory (no waveforms shown). In a follow-up study, Lempert et al. (1950) could record responses—again no waveforms were shown—in 13 out of 32 ears. They also suggested the placement of the electrode through the eardrum onto the promontory as a feasible non-surgical technique. Then the Ruben era ensued when Ruben et al. (1959, 1960) recorded CMs from the round window with clear waveforms for moderate level sounds produced by tuning forks and human whistles. The feasibility of ECochG as a diagnostic method was advanced when Ruben et al. (1961) recorded the first compound action potential (CAP) with clear N_1_ and N_2_ components from the round window. They quantified especially the N_1_ latency to a click and found that it was longer at threshold in humans compared to cats. Ruben et al. (1962) extended their recordings to children with serious verbal communication difficulty, seriously impaired speech, and who gave no subjective evidence of hearing. Ruben and Walker (1963) recorded CAPs in Ménières’ disease and found them similar to those in other humans when recorded from the round window. Finally, Bordley et al. (1964) reviewed the results obtained by the Ruben group in 63 patients, among those ECochGs obtained before and after stapes surgery. Clear nearly noise free N_1_N_2_ waveforms were shown. Ronis (1966) also presented some results pre- and post-stapedectomy, and suggested the use of the N_1_ latency as a “valid index of improved sound conduction”. Reviewing his work, Ruben (1967) mentioned three important topics in ECochG: (1) the correlation of physiological and psychoacoustic properties; (2) the investigation of certain diseases; and (3) the objective diagnosis of individual cases of deafness. The Ruben era of using ECochG was characterized by improved CM measurements and clear CAP recordings at moderate-to-high stimulus levels. It was still impossible to measure the CAP near the subjective threshold, and ECochG as based on round-window recording was still an operating room technique.

### Non-Surgical Period

The non-surgical period, started in 1967—time points refer to Figure 1—with the first publications by two groups one in Tokyo, Japan, led by Nobuo Yoshie and Toru Ohashi (Yoshie et al., 1967; Yoshie, 1968; Yoshie and Ohashi, 1969), and the other in Bordeaux, France, led by Michel Portmann and Jean-Marie Aran (Portmann et al., 1967; Aran and Le Bert, 1968; Aran, 1971; Portmann and Aran, 1971). The Japanese group started with extra-tympanic (ET) recordings but later on also used trans-tympanic (TT) recording as well. The Bordeaux group only used TT recordings. The period ending 1978 reflects in part the fairly large output from the Leyden group in Netherlands starting with Eggermont et al. (1974) and only using TT recording. The period ending 1984 signaled a starting interest in applying ECochG to the diagnosis of Ménière’s disease. After a reduction in the output in the period ending 1990, things picked up again in the following 12 years with a surge of papers on improving the use of ECochG in the diagnosis of Ménière’s disease. This was followed by a slump, both in the total number of ECochG articles and in the Ménière articles, potentially by disappointment in the clinical usefulness of the ECochG (Nguyen et al., 2010). The last six and a half years again show a steep incline in interest for ECochG fueled by its use in auditory neuropathy, its revival in Ménière’s disease following better diagnostic use of all information in the recorded waveforms, as intra-operative tests for cochlear implantation, and using the cochlear implant electrodes to perform multi-channel ECochG.

## Basic Principles

### Hair Cell Potentials

Both inner hair cells (IHC) and outer hair cells (OHC) generate receptor potentials in response to sound (Russell and Sellick, 1978; Dallos et al., 1982). It has long been known that compound responses from the cochlea reflecting these hair cell potentials can be recorded at remote sites such as the round window, tympanic membrane or even from the scalp, and can be used clinically. These responses are called the CM and the summating potential (SP). The CM is produced almost exclusively from OHC receptor currents and when recorded from the round window (RW) membrane is dominated by the responses of OHCs in the basal turn. The SP is a direct-current component resulting from the non-symmetric depolarization-hyperpolarization response of the cochlea, which can be of positive or negative polarity, and is likely also generated dominantly by the OHCs (Russell, 2008).

#### The Summating Potential

Dallos (1972) recorded, using electrodes in the scala vestibule and scala tympani, a differential component (DIF SP) and an overall component (AVE SP) of the cochlea. The DIF SP represents the DC-shift between scala vestibuli and scala tympani, and the AVE SP represents the DC shift of the entire cochlea relative to the neck muscle potential. The AVE SP is positive at the site of maximum stimulation and negative elsewhere in the cochlea. For a round-window recording from the guinea pig, one nearly always measures a positive SP^+^ when stimulation occurs with high-frequency, high-level tone bursts. This, therefore, may be compared to the AVE SP recorded from the first turn.

**Figure 2:**
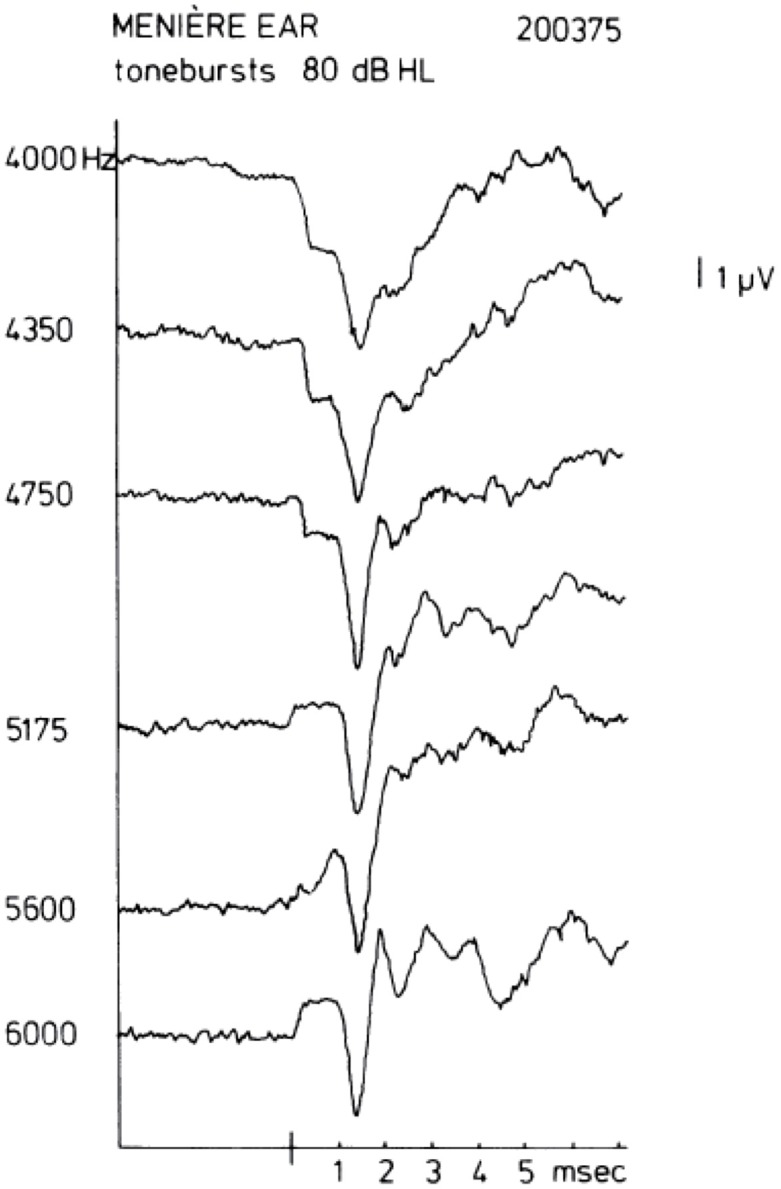
**Transition of a negative to a positive summating potential (SP^+^) with increase in tone burst frequency.** In some ears, a quite sudden change in the sign of the SP may be observed. In this example, a change occurs between 4750 Hz and 5175 Hz. This type of transition from SP^−^ to SP^+^ typically occurs between 4 kHz and 8 kHz. From Eggermont (1976c).

In human ECochG recordings from the promontory, the SP is most often negative in polarity (SP^−^). Sometimes, a change of sign is observed when the frequency of the tone burst is increased while keeping the intensity the same. A sequence of this kind is shown in Figure 2 taken from Eggermont (1976c). A comparable polarity transition occurring between 4 kHz and 8 kHz was shown in Dauman et al. (1988). For this ear of a patient with Ménière’s disease, a distinct SP^−^ is observed for a stimulus of 4000 Hz at an intensity of 80 dB HL; an increase in the tone burst frequency to 4350 or 4750 Hz leads to a clear drop in the SP^−^ amplitude. A further increase in frequency to 5175 Hz gives an SP^+^, whose magnitude increases slightly when the frequency is raised further. The same type of change is observed from the AVE SP recorded from the guinea pig’s first cochlear turn (Dallos, 1972), where, at an intensity of 60 dB SPL, the AVE SP is typically negative for frequencies up to 3000 Hz, about zero at 6000 Hz, and positive for higher frequencies. This could explain the changes if the promontory recorded SP is considered a mix of positive and negative AVE SPs generated in the basal turn depending on the resistance paths through the promontory into the cochlea and via the round window, i.e., the electroanatomy of the recording site.

**Figure 3:**
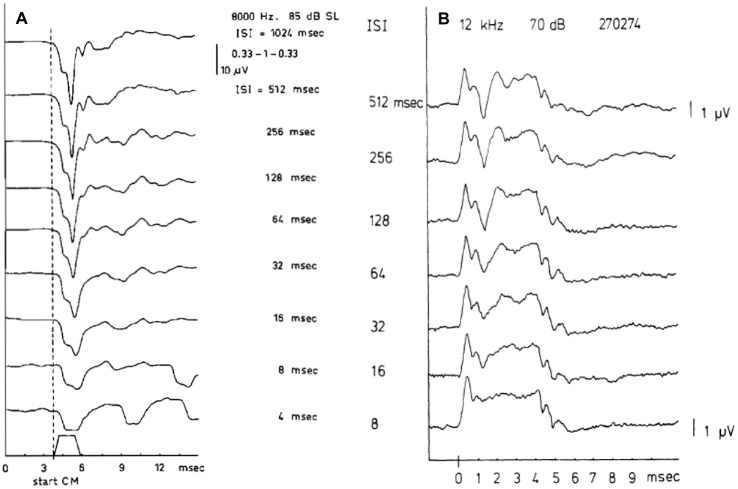
**(A,B)** SP and compound action potential (CAP) waveforms as a function of the interstimulus interval (ISI). The SP, being a pre-synaptic potential, does not show the phenomenon of adaptation as the CAP does. When the ISI value is lowered the CAP amplitude decreases but the SP amplitude remains constant. Panel **(A)** shows at an ISI of 4 ms, only the SP^−^ remains and closely resembles the stimulus envelope. From Eggermont and Odenthal (1974a). Panel **(B)** shows a combination of SP^+^ and SP^−^ in one recording. In ears showing a transition from SP^−^ to SP^+^ as described in Figure 6, for high frequencies a quite peculiar phenomenon may be observed. It appears as an SP^+^ followed after some latency by a smaller SP^−^ thus forming an early positive peak, which is persistent to low intensity levels. From Eggermont (1976c).

As a pre-synaptic potential the SP will not be affected by adaptation. Increasing the repetition rate of the stimuli will isolate the SP^−^ from the CAP as illustrated in Figure 3A. This works for both the SP^+^ and SP^−^, but shows surprisingly in this example that the SP^+^ may consist of a sharp transient and a sustained part (Figure 3B). This likely results from a combination of a short latency SP^+^ followed by a superimposed slightly longer latency SP^−^, both having a duration equal to the tone burst, and the SP^−^ originating from a slightly more apical region. We noted that this effect persists at least down to 55 dB HL. That this SP^+^/SP^−^ complex is not of neural origin is demonstrated by its persistence at short interstimulus intervals (ISI; Figure 3B). The CAP disappears nearly completely at an ISI of 8 ms, but the SP combination remains. The finding that the SP^+^ occurs more often in Ménière’s disease (Eggermont, 1976c; Dauman et al., 1988) could be caused by a changed electroanatomy, potentially attributable to an endolymphatic hydrops.

#### The Cochlear Microphonics

I had never much faith in the clinical use of the CM (Eggermont, 1976c), amplified by the fact that in Ménière’s disease the CM amplitude for 85 dB HL tone bursts was up to CAP thresholds of 70 dB HL independent of hearing loss (Eggermont, 1979a). However, as a consequence of the decisive use of CM in the diagnosis of auditory neuropathy (see “Auditory Neuropathy” Section) it is time to take a new look. The CM is an electric response that can be recorded from almost anywhere in the cochlea and from the cochlear surface (e.g., the round window), as first demonstrated by Wever and Bray (1930). Early on, Tasaki et al. (1954) showed that CM to all frequencies might be recorded with differential electrodes from the first turn in the guinea pig cochlea. Experiments in kanamycin-intoxicated guinea pigs, which destroys the OHC, showed that the CM produced by the IHCs was about 30–40 dB less sensitive than that generated by the OHCs (Dallos and Wang, 1974). However, absent CM is not an absolute indicator of non-functional OHCs, as Liberman et al. (2002) have shown in mice lacking prestin, the distortion-product oto-acoustic emissions (DPOAEs) are elevated to correspond to the hearing loss, whereas the CM is not significantly reduced compared to normal controls. The CM recorded at the promontory or in the ear canal thus arises primarily from OHCs in the more basal portions of the cochlea, while the apical regions make a negligible contribution to its generation (Johnstone and Johnstone, 1966; Patuzzi et al., 1989; Withnell, 2001). However, CM as recordable from the promontory may not only be generated in the basal turn and may for low frequencies also include neural contributions (Chertoff et al., 2012, 2014; Kamerer et al., 2016).

**Figure 4:**
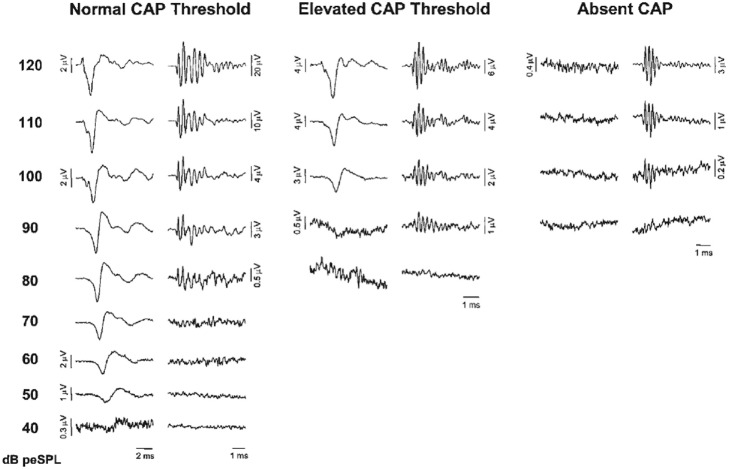
**ECochG recordings obtained from three representative ears showing normal CAP threshold, elevated CAP threshold and the absence of neural response at maximum stimulation intensity (clicks, 120 dB p.e. SPL).** CAP and cochlear microphonics (CM) traces were obtained by the classic procedure of averaging recordings to condensation and rarefaction clicks. Note the ringing of the click-evoked CM. From Santarelli et al. (2006).

Santarelli et al. (2006) recorded CM, SP and CAPs using TT ECochG in 502 normal hearing subjects and with varying degrees of sensorineural hearing impairment, and in 20 auditory neuropathy patients. They distinguished three categories (Figure 4), those with a normal CAP threshold in which case the CM to clicks is detectable to about 80 dB peak equivalent sound pressure level (p.e. SPL) (~50 dB HL), those with an elevated CAP threshold often accompanied by a CM with similar threshold, and those without CAP, where the CM might indicate functioning OHCs, as in auditory neurpathy. Santarelli et al. (2006) found that CM was almost always detected when recording TT ECochG in ears with varying degrees of hearing impairment or even with profound hearing loss, and thus, in the presence of extensive OHCs loss (Eggermont, 1979a; Arslan et al., 1997; Schoonhoven et al., 1999). Even in the 202 ears of children (mean age 2.6 ± 4.2 years) with no CAPs recorded at 120 dB peSPL, the CM was always detected albeit with elevated threshold (99.1 ± 7.9 dB p.e.SPL, compared to 41.1 ± 9.5 dB in normal controls) and reduced amplitude (7.5 ± 9.7 μV, compared to 29.1 ± 33.1 μV in normal controls). According to Santarelli et al. (2006) “this finding challenges the widely accepted view that the CM is strictly related to OHC electrical activity with only a minor contribution from IHCs”. An important finding was that the presence of central nervous system pathology and normal hearing thresholds seemed to enhance CM amplitude compared to normal hearing ears. This amplitude enhancement was often accompanied by prolonged CM duration, albeit that this duration enhancement was also observed in about half of completely normal ears (Gibbin et al., 1983; Liu et al., 1992; Santarelli et al., 2006). The amplitude enhancement was attributed to a dysfunction of the medial efferent system through a reduced inhibitory influence on OHCs, leading, in turn, to enhanced cochlear amplification. Santarelli et al. (2006) also compared DPOAEs with CM in the same ears with a wide range of CAP thresholds and found the presence of DPOAEs “a more sensitive indicator of hearing threshold preservation than CM amplitude”.

#### Interlude: Transtympanic vs. Extratympanic Recording

Of interest for addressing this choice I present four prospective studies that compared TT and ET ECochG in the same ears, and in two cases by simultaneous recording. Mori et al. (1982) concluded that TT showed higher amplitudes but the same latency as ET. Noguchi et al. (1999) confirmed this and also found that TT and ET had same threshold detection levels, and same slopes for the CAP amplitude-intensity functions. Of relevance for diagnostics of Ménière’s disease, to be reviewed later, is that TT recordings often show positive summating potentials (SP^+^) for high-frequency tone bursts and negative summating potentials (SP^−^) for lower frequencies, whereas in ET only SP^−^ were recorded (Mori et al., 1982). No significant difference in the SP/CAP ratio was found between TT and ET recordings (Roland et al., 1995). Schoonhoven et al. (1995) made simultaneous ET and TT recordings in 30 patients with various types and degrees of cochlear hearing loss. They found that ET responses were reduced in amplitude with respect to TT responses by a factor of 0.43 on average. ET and TT latencies were identical. This suggests that when the hearing loss is not too large both recording methods are equally applicable. Modifying the ET technique by using two identical high-impedance electrodes on the tympanic membrane (active) and as a reference in the ear canal, resulted in a signal to noise increase by >2.6 dB (Kumaragamage et al., 2015).

### The Compound Action Potential

#### Phenomenology

**Figure 5:**
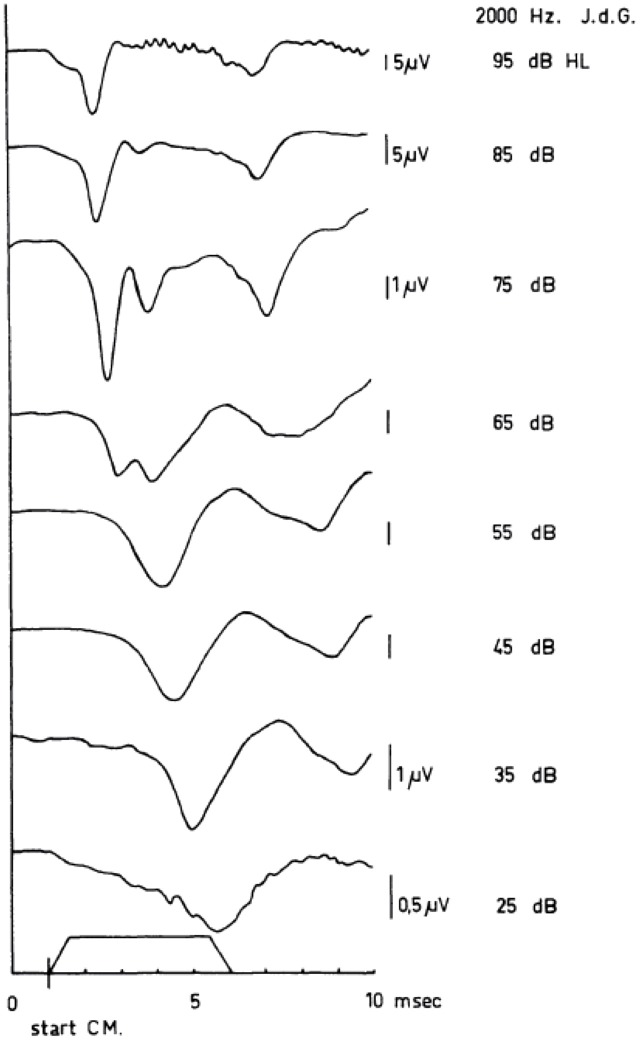
**Human cochlear APs as recorded in response to tone bursts (envelope shown near abscissa) between 95 dB HL and 15 dB HL.** In these series of APs, the sudden jump in the latency-intensity curves is illustrated. Note the appearance of a double-peaked N_1_/N_2_ complex at 65 dB HL and the difference between the latencies of the first negative wave at 65 dB HL and 55 dB HL. The scaling changes with intensity as indicated. From Eggermont (1976c).

I will introduce TT tone-burst ECochG with a typical intensity series of the CAP, obtained in a normal hearing subject for 2000 Hz tone burst stimulation (Figure 5). In this series of responses, an interesting transition takes place around 65 dB HL. If the intensity of 65 dB is taken as a starting point for the analysis, it is evident that an increase in intensity leads to a relative increase of the N_1_ peak of the CAP with respect to the N_2_, whereas lowering the intensity favors the second peak over the first (Eggermont and Odenthal, 1974a,b; Eggermont et al., 1974; Eggermont, 1976c). A similar bifurcation around 55–60 dB HL for click responses was reported by Yoshie (1976). A detailed analysis of the same phenomenon obtained in response to a 2 kHz half-sine wave stimulus from the external ear canal was carried out by Elberling (1973). He presented stimuli in consecutive 2.5 dB steps over the intensity range of 72.5–95 dB p.e. SPL. The two peaks were of about the same magnitude at 85–90 dB p.e. SPL, which corresponds to about 65–70 dB HL. It is tempting to attribute these two peaks to contributions from populations of auditory nerve fibers (ANFs) with low and medium thresholds respectively (Bourien et al., 2014).

**Figure 6:**
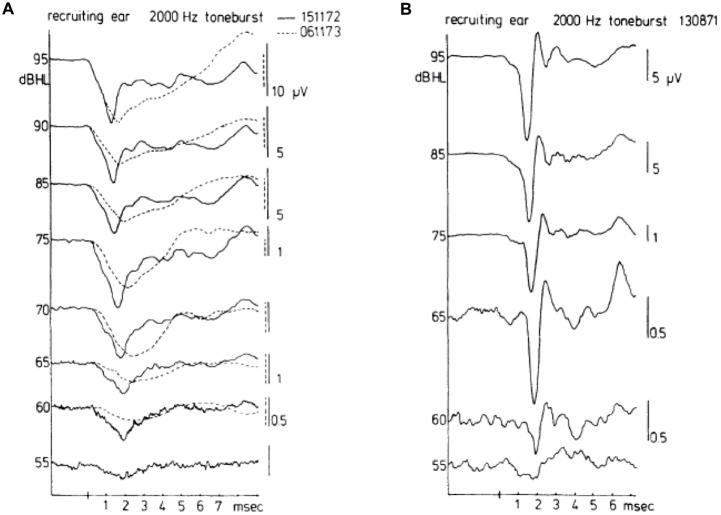
**Waveforms of CAPs in recruiting ears.** These waveforms may be either of the broad type **(A)** or of the biphasic type **(B)**. The diphasic type in this case was recorded for a cochlea with hearing loss to asphyxia at birth. The absence of a bimodal N_1_ complex and the consistent short latencies along with an abrupt amplitude decrease draw a distinction between normal (see Figure 5) and recruiting ears. The broad type was recorded in a Ménière ear. There was an interval of about a year between the times (dates in **A** top right) when the two sets of waveforms were recorded for the Ménière ear; quite dramatic changes are noted in the about 1 year time difference. From Eggermont (1976c).

A contrasting series of CAP waveforms for two types of sensorineural hearing loss with recruitment, resulting from: (a) Ménière’s disease; and (b) neonatal asphyxia, is shown in Figure 6, again for stimulation with a 2000 Hz tone burst. It is noted that in the neonatal asphyxia waveforms only the early N_1_ is present (see Figure 5), whereas in the Ménière ear the CAP is much broader and dominated by the relatively large and long lasting SP (see “The Cochlear Microphonics” Section).

**Figure 7:**
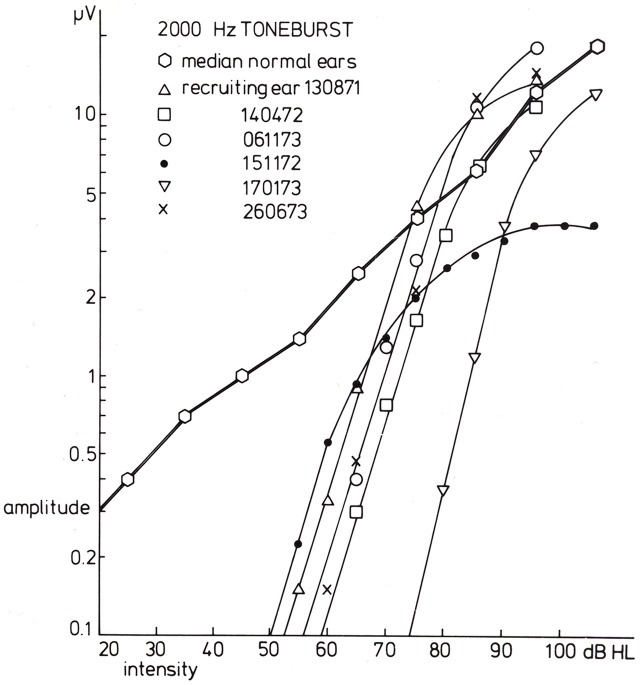
**Input-output curves for recruiting ears in response to 2000 Hz tone burst stimulation.** For six recruiting ears, the input-output curves are drawn. The data for the three series of CAPs shown in the former figure are indicated by triangles (the diphasic type) and open and filled circles (the Ménière ear). A common phenomenon for all curves is that the slope for amplitude values below, e.g., 1.5 μV is essentially the same and much larger than that found in normal ears. The median input-output curve is based upon data for 20 normal ears. From Eggermont (1977).

In case of loudness recruitment one often (but not always; Eggermont, 1976c) observes a steep increase in the amplitude of the CAP with stimulus level as illustrated in Figure 7. This shows a series of typical input-output curves for Ménière ears with the median curve obtained in 20 normal ears. All ears show the increase in steepness compared to the median control amplitude-level function (for which the threshold at the 0.1 μV level was at 0 dB HL). This mimics the steeper increase of loudness with increasing sound level.

**Figure 8:**
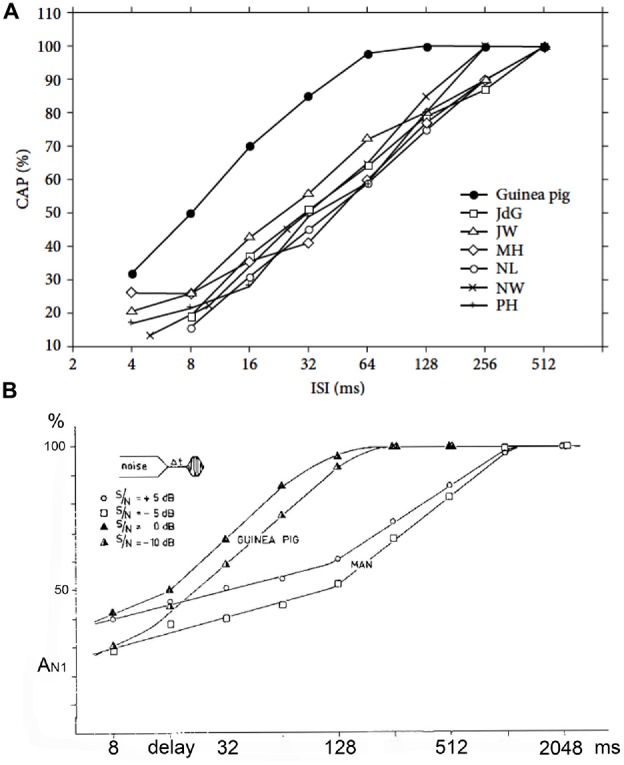
**(A,B)** Adaptation and forward masking of the CAP. **(A)** The amplitude of the CAP depends on the ISI. For six normal human cochleas, the relative decrease in amplitude is shown and compared to the mean for guinea pigs at a comparable stimulus level and shows a clear difference. The 50% relative amplitude point is found at a time about four times longer in humans than in the guinea pig. ISI, inter-stimulus interval. From Eggermont and Odenthal (1974a). **(B)** The relative CAP amplitude value in a forward-masking experiment as a function of the delay between the end of the white-noise masker and the tone-burst. In this experiment a 400 ms white-noise masker precedes a shore tone-burst. The CAP amplitude in response to this tone-burst depends on both the time (6t) after the masker and the intensity ratio between masker and tone-burst. In the human it takes about 1 s for full recovery from masking; in the guinea pig this value is about four times smaller. Δ*t*, post-masker delay. From Eggermont and Odenthal (1974b).

Early on it was noted that the adaptation and post-masking recovery in human CAPs was clearly different from animal data (Coats and Dickey, 1972; Eggermont and Spoor, 1973; Eggermont and Odenthal, 1974a,b). This is illustrated in Figure 8. Here we compare the adaptation of the CAP amplitude for stimulation with tone-burst trains of various ISI and in the recovery from forward masking as a function of post-masker delay (Δt) in guinea pigs and humans. Coats and Dickey (1972) found that the post masking recovery of click loudness in their ECochG participants was nearly complete at *Δt* = 100 ms, which compared well with the animal electrophysiological results, but not with the human ECochG. This suggests that CAPs, which depend on neural firing synchrony, do not reflect loudness measures.

#### The Composition of the Compound Action Potential

This topic got a lot of attention in the 1970s, where investigators aimed at understanding how to interpret human ECochG recordings. Early on, Gasser and Grundfest (1939) had used convolution to predict the waveform of the CAP evoked by electrical stimulation of the saphenous nerve of the cat from the distribution of nerve fiber diameters (resulting in a latency distribution) and a hypothetical individual fiber unit response. Twenty years later, Goldstein and Kiang (1958) pointed out that, under the assumption that unit responses add with equal weight to the recording electrode, the CAP-waveform could indeed be expressed as a convolution integral:

$$CAP\left(t\right)\;=\;N\int_{0}^{t}s\left(\tau \right)a\left(t-\tau \right)d\tau$$

where *N* is the number of nerve fibers, *s(t)* the latency distribution function and *a(t)* the unit response. A unit response, recorded from a nerve end, will be normally diphasic in shape and this has been postulated for the auditory nerve by Teas et al. (1962), de Boer (1975) and Elberling (1976a,b) and first demonstrated by Kiang et al. (1976). The convolution is allowed under the conditions of statistical independency of the individual contributions. When using a click as a stimulus, the latency distribution function may be considered reflecting the envelope of the impulse response function of the peripheral hearing organ. For individual fibers such impulse response functions may be obtained from the cross correlation between the nerve fiber response and a white noise stimulus evoking them (de Boer, 1969).

Investigating the single nerve fiber firing pattern for non-click stimuli will result in a modified weighting function s*(t) which may be found by convolution of the true impulse response and the stimulus envelope. A second convolution of the new s*(t) with the unit response *a(t)* will then give the CAP to this new stimulus (de Boer, 1975) after summation over all contributing units. In a practical situation, either in modeling or analyzing, the number of contributing units has to be restricted. This may be done by forming groups of nearly equivalent units. It might thus be useful to divide the cochlear partition into small regions about 3 mm long (corresponding to about half-an-octave in frequency) and study the narrow-band CAPs (NAPs) evoked on these small segments. Since the human cochlea is innervated by about 25,000 (Hall, 1967) to 31,000 (Rasmussen, 1940) afferent nerve fibers, such a 3 mm segment is assumed to comprise about 2500–3100 individual nerve fibers. The thresholds of the fibers in each segment are supposed to be approximately distributed in the same way across low-, medium- and high-threshold ones (Kiang et al., 1965; Rutherford and Roberts, 2008; Bourien et al., 2014).

**Figure 9:**
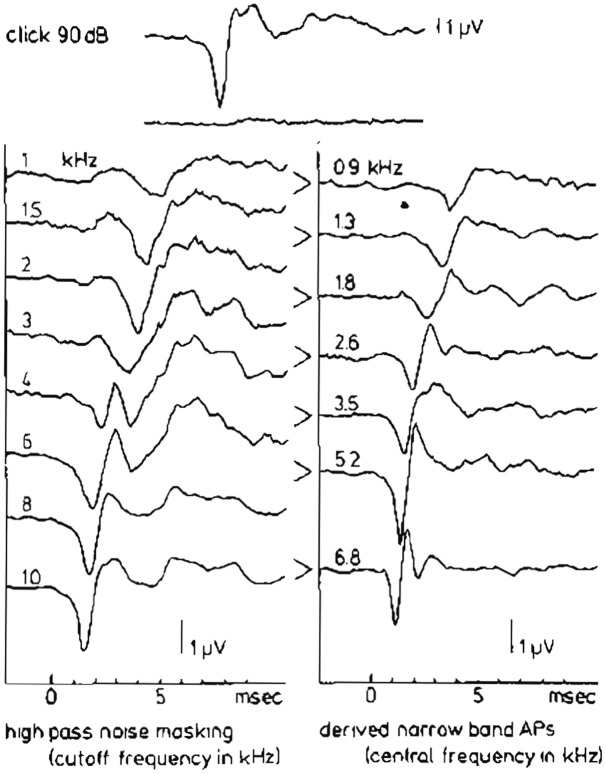
**High-pass noise masking and the derivation of narrow band APs (NAPs) in humans.** The upper two traces show the whole nerve CAP for a normal ear in response to a 90 dB p.e. SPL click and reflect the situation where just complete masking by wide-band noise occurs. On the left hand side the effect of high-passing the noise at successively higher cut-off frequencies can be seen. Subtraction of two subsequent CAP’s results in the set of narrow-band CAP’s in the right-hand side. From Eggermont (1979c).

Teas et al. (1962) introduced an experimental technique for such a separation of the CAP recorded from the guinea pig cochlea into about 10 NAPs. A high-pass noise-masking stimulus with a number of discrete high-pass cut-off frequencies was used. Subtracting CAP responses obtained in the presence of high-pass noise with cutoff frequencies being $\frac{1}{2}$ octave apart, results in NAPs, which can be assigned to particular narrow-band segments each characterized by a central frequency (CF). This technique has first been used in human ET ECochG by Elberling (1974) for the analysis of click-evoked CAPs. Later on this method was applied by Eggermont (1976d, 1979b,c) using TT recording of responses to click and tone burst stimuli to elucidate the frequency specific character of these types of stimulation. An example of such a separation of the CAP into NAPs for the human cochlea upon click stimulation is shown in Figure 9. The click intensity is 90 dB p.e. SPL, and the NAPs are essentially diphasic in shape and their latencies range from 1.4 ms to 5.8 ms. The CAP latency is 1.4 ms and is therefore mainly dominated by the most basal contributions, due to the diphasic waveforms the contributions from segments with lower CFs tend to cancel each other and are therefore not seen in the CAP. It seems appropriate to use narrow-band waveform for the highest CF, with the shortest duration, as an estimate of the unit response. It is noted that double peaked CAP responses as shown for 2 kHz tone burst in Figure 5, and observed here for 4 kHz high-pass noise masking of the click evoked CAP, are not the result of changes in the NAP waveforms but result from changes in the cancellation of responses from different CF regions.

**Figure 10:**
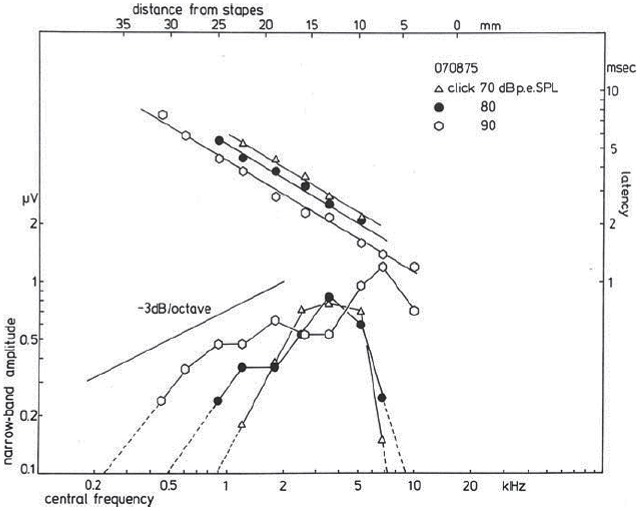
**Narrow band response parameters as a function of central frequency (CF).** For clicks of 70, 80, and 90 dB p.e. SPL, narrow band amplitudes are shown as a function of distance from the stapes; it is observed that for the highest intensity the amplitudes decrease by about 3 dB/octave. Lowering the click intensity results in a decrease for contributions from both the apical and basal part of the cochlea, while the central part still contributes the same. The latency data show an exponential dependency on the distance from the stapes, and a definite effect of stimulus intensity thereupon is noted. From Eggermont (1976c).

A plot of the NAP-amplitude (negative deflection only) as a function of the CF, which may be related to distance from the stapes (Greenwood, 1961; von Békésy, 1963), shows for a click level of 90 dB p.e. SPL (Figure 10) a gradual increase in amplitude for higher central frequencies. For lower click intensities, the contributions from both the high- and the low-frequency side rapidly decrease, while the central region (about 3 kHz) still contributes the same. For relatively low intensities, the activation area seems to be reduced to a more narrow frequency-selective region likely related to the external ear canal and middle ear resonances, which favor the parts in the spectrum around 2–3 kHz, where the human ear has its greatest sensitivity. In normal ears, and ears with high-frequency hearing loss, click evoked CAP thresholds will reflect the patency of this 2–4 kHz region.

More recently, in an elaborate and detailed study, Lichtenhan and Chertoff (2008) were able to estimate the number of ANFs, *N*, contributing to the CAP, as well as the post-stimulus time histogram summed across nerve fibers, *s(t)*, and unit response, *a(t)*, before and after TTS. They found that TTS resulted in: a broadening and decreased latency of *s(t)*, and decreased *N*. Their model unit response, *a(t)*, based on the whole nerve click CAP showed a lower oscillation frequency and more rapid decay. This could have been improved by using a high CF NAP. These results suggested that TTS causes fewer ANFs to contribute to the CAP and those that do are more basally located with lower response synchrony and more quickly decaying and lower frequency oscillations. Lichtenhan and Chertoff (2008) suggested that this type of analysis might be useful in quantifying the number and location of surviving ANFs in patients with hearing loss. Similarly, Earl and Chertoff (2010) fit the analytic CAP to gerbils with partial lesions of the auditory nerve. The model parameter *N* at high-stimulus levels was strongly correlated with normal nerve area suggesting, that it is a good predictor of auditory nerve survival. The model parameter N also seemed to be a better predictor of the condition of the auditory nerve than the conventional measure of CAP amplitude.

#### Validation of the Use of NAPs

Evans and Elberling (1982) validated the use of the high-pass noise masking technique by comparing single-unit recordings and CAP measurements in the cat under conditions of high-pass masking. They computed the NAPs in a cat and compared them with the CFs of single cochlear fiber responses contributing to these NAP regions. With one main exception, the conclusions drawn on the origin of the frequency components of the NAPs were found to be valid in the normal cat. The exceptions were fibers with characteristic frequencies below 1–2 kHz, where the high-pass masking derived location was less specific. Taking into account the low-frequency hearing range of the cat, which is shifted upwards with about 1 octave compared to humans, Evans and Elberling (1982) predicted that the high-pass masking technique would be valid in normal humans for frequencies down to 0.5–1 kHz. This in effect validated the use of the latency distribution function.

Further experimental evidence for the applicability of the NAP technique in pathological cochleas came from recordings in normal and noise-exposed guinea pigs (Versnel et al., 1992), which looked at the validity of using the same unit response along the CF range and in normal vs. hearing loss ears. They used a technique pioneered by Kiang et al. (1976) involving spike-triggered averaging of round window “noise”. In that way one can estimate the unit response for units with CFs corresponding to locations along the cochlear partition. Their findings in normal cochleas confirmed the earlier data from Prijs (1986), namely that the unit response was diphasic and had a fairly constant amplitude of about 0.1 μV. In noise-exposed cochleas, waveform, latency and amplitude of the negative component of the unit response remained unchanged.

Delays estimated from NAPs have recently been used to generate chirps, which synchronize auditory nerve discharges along the length of the cochlea and yield larger amplitude CAP responses than clicks, presumably due to greater ANF synchrony along the cochlear partition (Chertoff et al., 2010).

### Diagnosis Based on the Waveform of the Compound Action Potential

Portmann and Aran (1971) were the first to point to a potential diagnostic use of the click-evoked CAP waveform. They distinguished four typical response patterns: the normal response, the recruiting response (not unlike that in Figure 6B), the broad or prolonged response often seen in Ménière ears (see Figure 6A), and the abnormal response, which showed an initially positive SP (Figure 3B). Yoshie (1976) also paid attention to the abnormal waveforms found in Ménière ears and resulting from SP^+^ and/or SP^−^ interaction with the CAP. Much attention was paid on the so-called low- and high-amplitude and latency functions with a cross-over point at the bifurcating CAP waveform (see Figure 5).

**Figure 11:**
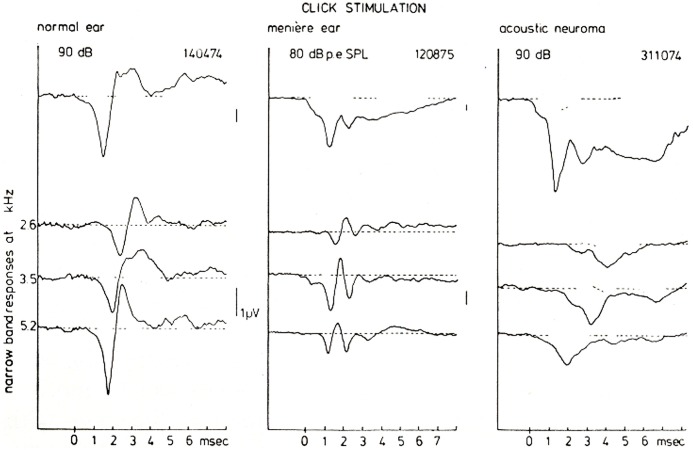
**CAP and NAP waveforms for a normal ear, a Ménière’s ear and a neuroma ear.** As has been observed consistently in many cases there is a typical Ménière’s and acoustic neuroma type of AP waveform which is very distinct from normal. The distinction between both pathologies on basis of the CAP-waveform, however, in general presents some difficulties. A narrow-band analysis shows that the individual NAP-waveforms are different for all three hearing states, which may be of help in further diagnosis but also provides an insight in the location of the disturbance. From Eggermont (1976a).

However, these typical waveforms and their presumed reflection of the underlying disturbances in the peripheral hearing organ can be studied more insightfully by using the narrow-band response derivation. Figure 11 shows such a (CF-restricted in these illustrations) narrow-band analysis for a normal ear, a Ménière ear and for an ear affected by an acoustic neuroma (vestibular Schwannoma). For the normal ear the narrow band responses at the three central frequencies shown are essentially biphasic in shape (see Figure 9). Since in this basal part of the cochlea the traveling wave velocity is around 20 m/s (Eggermont, 1976d), these 3 mm wide narrow bands are traversed by the traveling wave in about 0.15 ms. One may say therefore that these single nerve fibers will fire in nearly perfect synchrony. This implies that for the most basal part of the cochlea the NAP reflects the unit-response waveform contribution to the CAP.

For the Ménière ear the recorded CAP is dominated to a large extent by the relatively large negative SP. The narrow band analysis, however, shows an additional feature, namely that the unit contribution is composed out of two biphasic waveforms with a delay of about 1 ms. This may point to repeated firing by the fibers in the indicated narrow-bands in response to the same click. This fact may contribute to the over-recruitment often observed in Ménière ears but this will need a more detailed study.

The acoustic neuroma ear shows essentially the same type of broad CAP waveform as found in the Ménière ear (see also Aran, 1971). However, the SP is relatively smaller than the CAP and seems not to account for the broadening of the CAP in the same way as in the Ménière ear. High-pass masking shows that the NAPs are monophasic in this situation. The addition of the NAPs therefore does not produce cancellation of activity after the onset of the CAP as found in normal and Ménière ears, but instead produces a broad CAP. In this situation the NAP waveform may reflect a change in the unit contribution as a result of nerve conduction block due to the presence of the tumor (Beagley et al., 1977).

The mechanisms that produce these striking differences in NAPs seem very useful in diagnosis. Especially the close similarity of the CAP waveforms for the Ménière ear and the neuroma ear is completely removed when looking at the narrow band responses.

## Clinical Applications

### Objective Audiograms

Sample objective audiograms for frequencies of 500 Hz to 8 kHz obtained with TT tone burst ECochG were first shown in Eggermont et al. (1974). For a more restricted frequency range, Yoshie (1973) performed a regression analysis in 56 patients between TT CAP thresholds for tone pips with the audiometric thresholds for the frequencies 2, 4, and 8 kHz. The regression lines showed slopes that ranged from 0.75 (2000 Hz) to 0.83 (4000 Hz) with correlation coefficients very near 0.90. Almost all of the points in his scattergram were within ± 15 dB from the regression line, suggesting good clinical application.

In a group of 96 patients in which behavioral audiometry was available, Spoor and Eggermont (1976) compared the audiogram with TT ECochG tone burst evoked CAP thresholds. Given an ECochG threshold, the practical question concerns the prediction of the subjective threshold. Regression analysis showed that the slope of the regression line was close to unity for each frequency. For 1, 2 and 4 kHz, the mean difference between ECochG and subjective measures was 0 dB. At 500 Hz, the mean difference between the ECoG and the subjective thresholds was about −10 dB, i.e., the subjective threshold is 10 dB higher than the ECoG threshold. At 8000 Hz this was the same, and the spread of ECochG thresholds at 500 Hz and 8 kHz was higher than at 1, 2 and 4 kHz. Standard deviations for the different frequencies varied from 7.5 dB to 11 dB, resulting in a 95% confidence level of 15–22 dB around the mean.

Schoonhoven et al. (1996) further investigated the relation between TT response thresholds for tone bursts with octave frequencies from 500 Hz to 8000 Hz and audiometric thresholds in 148 ears. Similar analyses of ET thresholds were reported for a subset of 30 ears in which TT and ET physiological responses were simultaneously recorded. They found that TT ECochG thresholds were highly correlated with audiometric thresholds. Linear regression analysis showed that audiometric thresholds might be predicted from physiological thresholds with an error in the estimate of 11 dB. ET ECochG permitted similar predictions but with a larger uncertainty of 16 dB. It appeared that ECochG thresholds increase slightly less with increasing cochlear dysfunction than do pure tone thresholds. They considered this a result of the different stimulus durations on which the two threshold measurements are based and the difference in temporal integration between normal and pathological ears.

Recent animal studies have provided an interesting alternative. Lichtenhan et al. (2013) described a novel technique to estimate low-frequency cochlear thresholds that uses the auditory nerve overlapped waveform (ANOW) response in the guinea pig. They showed that for frequencies of 700 Hz and below, ANOW thresholds were mostly 10–20 dB more sensitive than onset-CAP thresholds and 10–20 dB less sensitive than the most sensitive single-AN-fiber thresholds. The results show that ANOW can be used to objectively estimate thresholds at very low frequencies in a high frequency-specific manner. A subsequent study (Lichtenhan et al., 2014) demonstrated that in guinea pigs this ANOW response originates in the apex of the cochlea. This technique could potentially be used to assess very low frequency information more accurately than current ECochG procedures allow.

### Ménière’s Disease

#### The Importance of the Summating Potential for Diagnosis

The first report using non-surgical recording (TT) in Ménière patients (*N* = 22) was by Schmidt et al. (1974). They reported that the SP^−^ value, although often pronounced compared to the CAP amplitude, was almost the same as found in normal hearing ears, however distinctly larger than the SP^−^ amplitude observed in non-Ménière ears with high-frequency hearing loss. The first abnormal waveforms in a Ménière patient were shown in Schmidt et al. (1975) and later in Eggermont (1976a) and Odenthal and Eggermont (1976), all using TT ECochG. The use of the SP/AP amplitude ratio was first reported at an ECochG conference in 1974 organized by Bob Ruben in New York City and later published in Eggermont (1976b). However, I found this technique not useful for diagnosing individual patients. The mean SP/AP ratio in normal ears was level dependent and decreasing from about 0.3 at 95 dB HL to 0.07 at 55 dB HL, the upper limit was barely level dependent and about 0.45. In Ménière ears, the mean SP/AP value was nearly level (55–95 dB HL) independent at 0.35, with upper limits up to 0.6. In “hair cell loss” ears, the mean SP/AP ratio was strongly level dependent, from 0.25 at 95 dB HL decreasing to 0.06 at 75 dB HL. The upper boundary was around 0.6 at 95 dB HL and decreasing to 0.35 at 75 dB HL. Example waveforms contrasting a Ménière ear and a non-Ménière hearing loss ear, were shown in Figure 6. Note that the CAP amplitude in this study was taken from the level of the SP^−^ and not from the baseline, which would include the SP^−^ in case of tone burst stimulation, and thus reduces the calculated SP/AP ratio. For tone burst evoked responses measuring CAP amplitude from the SP level (either SP^+^ or SP^−^) seems to be the best procedure. For click evoked responses it is more difficult to assess the decaying SP level and here the least ambiguous way would be calculating amplitudes with respect to the pre stimulus baseline. Separate norms have to be established for ET and TT recordings.

Gibson et al. (1977) were more optimistic for SP use in diagnostic procedures by the observation of an “apparent widening of the SP/AP waveform”. They considered this as caused by an enlarged SP^−^, enhanced relative to the CAP, and “believed to be related directly to the presence of endolymphatic hydrops”. This was followed up Gibson et al. (1983) by a comparison of 32 normal, 40 sensory-loss ears, and 44 Ménière ears. They concluded that the diagnostic value would be increased if the SP amplitude was expressed as a percentage of the CAP amplitude, i.e., as an SP/AP ratio. In normal ears, the mean SP/AP ratio was 25% (range 10%–63%). In sensory damage, the SP/AP ratio was on average 13% (range 0%–29%), and in Ménière’s ears, the mean SP/AP ratio was 51% (range 29%–89%). In this series, an SP/AP ratio of 29% suggested a useful diagnostic dividing mark between the sensory damage and ears affected with Ménière’s disease. Note the large overlap in SP/AP range between normal and Ménière ears.

An extensive study of the SP^−^ in 112 patients with Ménière’s disease compared to 22 normal ears was carried out by Eggermont (1979a). He divided the Ménière ears in a low-threshold (≤50 dB HL) and a high-threshold (>50 dB HL) group. The SP^−^ values at a range of intensity levels (55–85 dB HL) were not significantly different from normal for the low-threshold group, whereas the high-threshold group showed significantly smaller SP^−^ amplitudes for 2, 4 and 8 kHz tone bursts. For 2 kHz the median amplitude value was independent of the hearing threshold up to 45 dB HL, and for larger losses there was a sharp decrease in the SP^−^ amplitude. The same phenomenon was found for 4000 Hz: up to 55 dB HL there was a slow decrease in the SP^−^ amplitude, and for higher threshold values a sharp loss. Thus the pattern at both frequencies showed a boundary value around 50 dB HL. The changes at 8000 Hz, however, seem more gradual relative to the amount of hearing loss, making separation artificial at this frequency. Eggermont (1979a) concluded that “in Ménière ears hearing losses up to about 50 dB are not related to changes in the hair cells, since the SP^−^ does not change, whereas the increase in the amount of hearing loss above 50 dB HL is paralleled by a loss in sensitivity of the SP and is therefore related to a functional loss of hair cells”.

Coats (1981) used clicks and ET recording, and measured both the SP^−^ and the N_1_ from baseline, which tend to make the SP/AP, in fact an SP/(SP + AP), ratio smaller. However, this may have a small effect when using clicks as the SP then is of small duration. Despite that Coats found that the SP/AP ratio for detection was 64%. I would not consider this a value useful for diagnosing individual cases. Goin et al. (1982) reported that the SP/AP was the most efficient diagnostic measure, with 62% of the Ménière’s group demonstrating abnormal ratios compared to 4% of the normal control group and 17% of the cochlear group. However, they did not report the “abnormal value” used. Kanzaki et al. (1982) using TT and ET ECochG found that “It was not possible to differentiate Ménière’s disease from sudden deafness on the basis of large SP/AP ratios alone. Such ratios were found frequently in both diseases”. Ferraro et al. (1985) used ET ECochG in 55 suspected Ménière patients and found that “the presence of hearing loss combined with aural fullness or pressure was the strongest predictor of an enlarged SP/AP ratio”. The Bordeaux group (Dauman et al., 1988) investigated the SP to 1, 2, 4 and 8 kHz tone bursts in 50 Ménière patients, 10 sensory loss patients and five normal hearing controls. They found that the mean SP amplitude was larger in the Ménière’s disease group for 1, 2 and 8 kHz compared to controls. However, the ears with larger negative SPs at low frequencies also had larger CAPs, measured from the level of the SP.

In a large series of studies Mori et al. (1987a) investigated differences between TT and ET ECochGs in the use of the SP/AP ratio for click and tone burst stimuli. The N_1_ amplitude included the tail of the SP. They found that the SP^−^/AP ratio at 80 dB nHL was higher for a click with the ET than with the TT method. The SP^−^ elicited by tone bursts of mid to low frequencies was found more stable in Ménière’s disease than SP^−^ elicited by a click (Mori et al., 1987b). An important observation was that there was no relationship between the ratio of the SP^−^ amplitude between both ears and the hearing threshold level at any frequency. In contrast, CAP amplitude ratio between both ears was significantly correlated (*r* = −0.419, *p* < 0.01) to the average hearing threshold level at 2–8 kHz, but not at 0.25–1 kHz (*p* > 0.05). This suggested that the increase in the SP^−^/AP ratio with the deterioration of the hearing at higher frequencies (Mori et al., 1987b) resulted from a decrease in CAP amplitude rather than an increase in SP^−^ amplitude (Mori et al., 1988; Asai and Mori, 1989). When the SP^−^/AP ratio threshold for abnormality was set at 0.43, they found that “ears with abnormal SP^−^ had a significantly worse hearing loss at high frequencies (2–8 kHz) than ears with normal SP^−^, whereas there was no significant difference in hearing loss at low frequencies (0.25–1 kHz) between both ears” (Mori et al., 1993).

The value of the SP/AP ratio that is considered indicative for Ménière’s disease varies between studies. We have seen that Gibson et al. (1983) favored a value of 0.29, whereas Mori et al. (1993) used 0.43. Koyuncu et al. (1994) used 0.33, Aso and Watanabe (1994) suggested 0.42, Pou et al. (1996) used a definite positive result for a ratio >0.5, and definite negative below 0.35. In a meta analysis of various studies, Wuyts et al. (1997) proposed an SP/AP ratio for click stimulation >0.35 using TT-ECochG, or >0.42 using ET-ECochG, as indicative of hydrops.

Specificity and sensitivity is important for any diagnostic test. Sass (1998) used TT ECochG in a group of 61 patients (61 ears) with the clinical diagnosis of Ménière’s disease and 15 patients (21 ears) with cochlear hearing loss of other etiologies, and 13 normal hearing subjects to assess the ability of the SP/AP ratio method to separate different cochlear disorders. Sass (1998) found a sensitivity of the click SP/AP ratio of 62% and a specificity of 95%. Inclusion of the 1-kHz burst-evoked SP amplitudes increased sensitivity to 82%, without changing specificity. Inclusion of the 2 kHz tone burst had no further effect on sensitivity or specificity. Sass et al. (1998) added the latency difference for condensation and rarefaction clicks, which was significantly larger in Ménière’s disease compared to normal and non-Ménière hearing loss (as was also found by Orchik et al., 1998; Ge and Shea, 2002), and found that “the sensitivity of TT ECochG, obtained by using measurements of SP/AP ratios and the SP amplitude at 1 kHz burst stimulation, increased from 83% to 87% by addition of the condensation-rarefaction shift measurement”. The specificity of TT ECochG obtained by this combination of variables was 100%.

Negative outlooks on the use of ECochG parameters in the diagnosis of Ménière’s disease started to emerge in the late 1990s. Levine et al. (1998) using CAP amplitude, SP amplitude, and CAP latency concluded that: “ECOG has limited value in the diagnosis of Ménière’s disease. It appears to correlate with the length of time patients experience symptoms and their audiometric findings. It was not correlated with the number of symptoms that the patient experienced at the time that the study was conducted”. This was echoed by Kim et al. (2005), who reported that abnormally elevated SP/AP ratios (>0.4) in definite Ménière’s disease were found in 66.7%. In less than definite Ménière’s disease this was only slightly lower by 52.7%, which was not significantly different. Consequently, based on the SP/AP ratio approximately 30% of those with definite Ménière’s disease would not be classified as having Ménière’s disease. Because of its lack of sensitivity, ECochG was considered not to play a decisive role in determining the presence or absence of Ménière’s disease. Gibson (2009) also found that click SP/AP measurements did not significantly differentiate between Ménière’s ears and non-Ménière’s ears. However, tone burst SP-amplitude measurements were found significantly different between the two groups, particularly for frequencies at 500 Hz, 1 kHz, and 2 kHz. Recently, Oh et al. (2014) reported that: “Statistically significant differences were not demonstrated in the SP/AP amplitude ratio or SP_area_/AP_area_ ratio between the definite Ménière’s, probable Ménière’s, overall Ménière’s, or control groups”. These less than positive findings were echoed by a questionnaire on the clinical utility of ECochG in the diagnosis of Ménière’s disease among members of the American Otological Society (AOS) and American Neurotology Society (ANS). It was found that “For approximately half of respondents, ECochG has no role in their clinical practice. ECochG was used routinely by only 1 in 6 respondents” (Nguyen et al., 2010). However, introducing more extensive measures such as SP/AP area ratio (Ferraro and Tibbils, 1999) in some studies appeared to increase the diagnostic sensitivity (Devaiah et al., 2003). By combining SP amplitude, SP area, SP/AP area ratio and total SP-AP area, sensitivity and specificity values increased to 92% and 84%, respectively (Al-momani et al., 2009). In contrast, Baba et al. (2009) found that the combination of these parameters as well as using SP/AP area alone did not have greater sensitivity than SP/AP amplitude ratios.

#### Evaluating Mechanisms of Ménière’s Disease

Dehydrating agents such as glycerol have been routinely administered since the report of Klockhoff and Lindblom (1966) to reduce the presumed endolymphatic hydrops in Meniere’s disease and improve hearing thresholds. Here are some of the pioneering ECochG studies. Moffat et al. (1978) tested 13 patients diagnosed with Ménière’s disease using TT ECochG during glycerol administration. Decrease of the SP^−^ was a common finding and occurred more often than threshold changes. Coats and Alford (1981) administered glycerol to 11 Ménière and 20 non-Ménière ears. ET-recorded SP amplitudes decreased, and 250–1000 Hz thresholds improved, and CAP amplitudes from the ears with Ménière’s disease also decreased after glycerol ingestion, but to a lesser degree. None of these changes were found in non-Ménière ears. Gibson and Morrison (1983) presented a single case study showing a large SP compared to the CAP, which after dehydration with glycerol showed a decrease in the SP and no change in the CAP so that the SP/CAP ratio became almost normal. Dauman et al. (1986) evaluated the “effect of orally administered glycerol on the SP and CAP amplitudes by means of automated recordings repeated every 5 min. SP values were remarkably constant in the control group. A decrease in SP absolute amplitude was observed in most patients with Ménière’s disease and some subjects with uncertain diagnoses, specifically at low frequencies.” Takeda and Kakigi (2010) evaluated 632 patients (727 ears) with vertigo/dizziness, of which 334 patients had a definite Ménière’s diagnosis. They found an enhanced SP in 56.3% of patients with Ménière’s disease, mostly where the disease duration was ≥2 years and/or the frequency of attacks was several times a year. Hearing improvement induced by the glycerol test did not produce a change in the SP/AP ratio—likely because both SP and AP increased or decreased together—and there was no significant difference between the glycerol test results and the incidence of an enhanced SP. Takeda and Kakigi (2010) suggested that the ECochG seems to indicate that the enhanced SP in Ménière’s disease might be caused by the malfunction of the hair cells, not by the displacement of the basilar membrane toward the scala tympani, i.e., not by an endolymphatic hydrops. Fukuoka et al. (2012) evaluated 20 patients with a 3T MRI scanner and ECochG after glycerol application. They found that ECochG was positive (SP/AP > 0.3) for hydrops in 15/20 patients and with MRI hydrops was detected in all but one of the patients.

The alternative to dehydration is the effect of salt loading, which was supposed to produce endolymphatic hydrops symptoms. After baseline ECochG studies, Gamble et al. (1999) administered 4 g of sodium chloride daily for 3 days to controls and Ménière’s disease patients. The control group of 13 healthy volunteers with normal baseline ECochG and pure tone audiometry was tested under similar conditions. Gamble et al. (1999) performed ET ECochG using alternating polarity clicks presented at a rate of 9.7/s at 95 dB nHL. A SP/AP ratio of 0.37 was considered the upper limit of normal. One or both ears in 38% of the patients in the study group with normal baseline SP/AP ratios and symptoms of inner ear fluid imbalance converted to abnormal. The mean SP/AP ratio of the control group for the conditions before and after salt-load was not statistically different (*p* = 0.48), whereas the difference in the mean SP/AP ratio in the study group after salt loading was statistically significant.

An animal experiment on the effects of endolymphatic hydrops, which is assumed to displace the basilar membrane towards the scala tympani and thereby increase the SP was carried out by Klis and Smoorenburg (1994). They used perfusion of the perilymphatic space with a hypotonic solution, which increased the SP and decreased the CAP amplitude, and corroborated the idea that static displacements of the basilar membrane indeed may underlie the enlarged SP and in particular the enlarged SP/AP ratio.

### Vestibular Schwannoma

One of the first studies using ECochG in the diagnosis of vestibular Schwannoma was by Morrison et al. (1976) who evaluated the findings in 56 surgically confirmed ears. They proposed that there are at least three separate criteria to be considered in reaching or strongly suspecting a diagnosis of such pathology. These are broadening of the CAP waveform (loss of the positive peak separating the N_1_ and N_2_), observation of a clear CM response, and presence of the CAP even when using stimulus intensities which are not audible in the patients’ affected ears. Beagley et al. (1977) explored in an animal study why the normally diphasic CAP changed into a monophasic one and attributed it to a neural block. This fits well with the monophasic NAPs often obtained in these tumors (see Figure 11).

**Figure 12:**
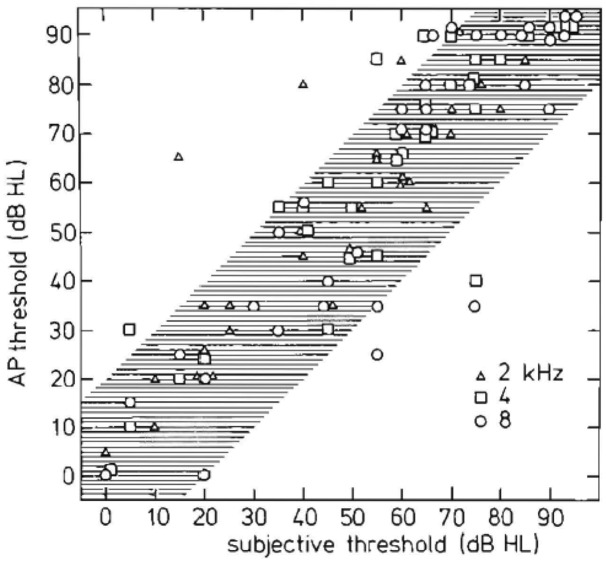
**Relationship between objective and subjective hearing thresholds.** Peripheral and central (subjective) measurements are similar except for a few ears. This similarity indicates that 8th nerve tumors usually produce a peripheral hearing loss (Eggermont et al., 1980).

In a large study Eggermont et al. (1980) compared the use of ECochG and auditory brainstem response (ABR) in the diagnosis of surgically confirmed vestibular Schwannoma in 45 patients. ECochG results provided evidence that, for hearing losses up to at least 60 dB HL, the origin is cochlear (Figure 12). We concluded that ECochG as the sole test for detection of vestibular Schwannoma appeared to be of limited diagnostic value. In combination with ABR, ECochG generally provided a clear N_1_ in cases where ABR wave I could not be detected, and so raised its diagnostic value.

**Figure 13:**
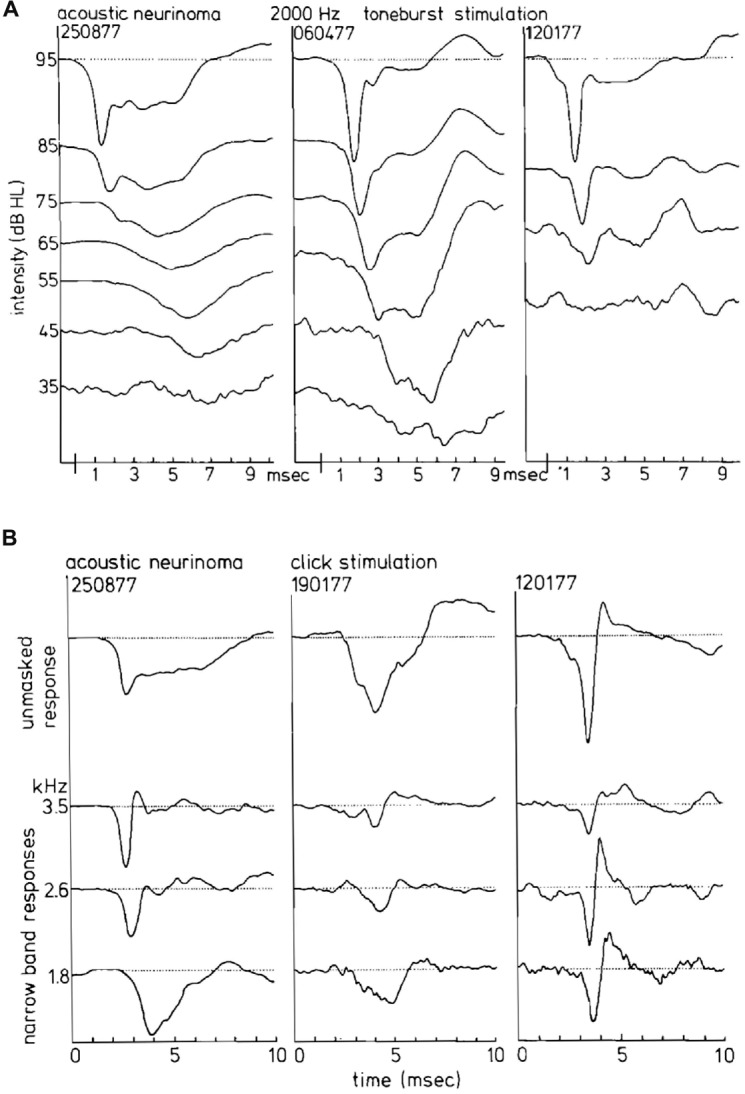
**(A)** CAP waveforms in response to 2 kHz tone burst stimulation in three ears with acoustic neurinoma. Depending on the individual case as well as on stimulus intensity, broad characteristic waveforms or nearly normal CAPs can be found. It appears that the CAP waveform is not consistently abnormal in acoustic neurinoma ears. **(B)** Narrow band AP waveforms in acoustic neurinoma ears. From dominantly monophasic NAPs in the left series to strictly biphasic narrow band responses in the right series, reminding us of a sensorineural hearing loss, the relationship to the CAP waveforrn is clear. From Eggermont et al. (1980).

CAP phenomenology in vestibular Schwannoma ears is distinctly different from normal ears and often also from ears with sensorineural hearing loss (Figure 13A). In 30% of the studied vestibular Schwannoma cases, Eggermont et al. (1980) found that the N_1_ latencies were longer than those of Menière’s disease. Whereas long CAP duration is found with use of tone burst stimulation, especially for 2 kHz, it does not occur in the NAP derivation (Figure 13B). Most cases with abnormally long N_1_ latencies also had monophasic narrow band contributions. In this situation, the usual canceling of positive and negative deflections leading to sharply peaked CAPs is lacking. The result is broad CAPs and abnormally long CAP latencies in the middle intensity range.

**Figure 14:**
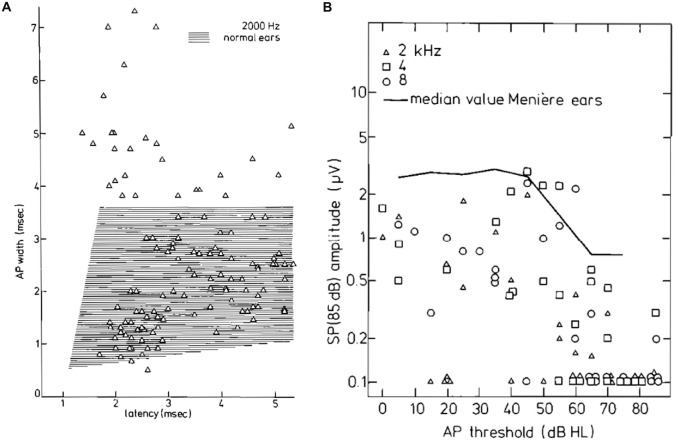
**(A)** CAP width-latency data for 2 kHz tone burst stimulation for the tumor ears. About 20% of the points are well outside the normal range. **(B)** SP amplitudes for 85 dB HL tone bursts as a function of the CAP threshold. Up to 50 dB HL thresholds, the SP amplitude appears stable; for higher hearing losses, the SP amplitude decreases and often the SP is absent. This trend is also observed in a large group of ears with Ménière’s disease whose median value is indicated. Moreover, the median values of tumor ears are smaller by a factor of at least 2. From Eggermont et al. (1980).

Correspondingly, the width of the CAP, resulting from the monophasic NAP contributions can be distinctly larger than in normal ears (Figure 14A), whereas the amplitude of the SP^−^ is clearly lower than in Ménière’s disease (Figure 14B). Thus the abnormally broad CAPs, especially those with short latencies (Figure 13A), are due to this NAP effect and not to a pronounced SP^−^, as in Menière’s disease.

Finally, Eggermont et al. (1980) found that the dominant effect of vestibular Schwannomas, causing a hearing loss, is on the cochlea probably resulting from interference with the blood supply. Because most ECochG parameters indicate a pure cochlear hearing loss without neural involvement, assessing the state of hearing at the peripheral site of the internal auditory meatus therefore has limited value in the differential diagnosis. An exception is when the CAP thresholds are much lower than the behavioral ones. This was later independently confirmed by Prasher and Gibson (1983).

## Current Interest

### Cochlear Implants

Telemetry capabilities became commercially available in 1998 (e.g., Shallop et al., 1999) for the measurement of the electrically evoked CAP (eCAP) from the auditory nerve in cochlear implant recipients. The eCAP is recorded via the intracochlear electrodes of the implant. Because the eCAP is a short-latency evoked potential, it overlaps with the stimulus artifact. All newer CI systems are equipped with two-way telemetry capabilities and artifact rejection that allow for measurement of electrode impedance and the eCAP. The eCAP is recorded as a negative peak (N_1_) at about 0.2–0.4 ms following stimulus onset, followed by a much smaller positive peak (P_1_) or plateau occurring at about 0.6–0.8 ms. The amplitude of the eCAP can be as large as 1 mV, which is much larger in magnitude than the CAP (up to 30 μV) recorded by TT ECochG in normal ears (Eggermont et al., 1974).

The ability to record high quality eCAP data was early on shown by Frijns et al. (2002). Their recordings showed clear N_1_ and P_1_ peaks with amplitude up to 400 μV, under the condition that there was at least one contact space between the stimulating and recording electrodes. They also found that responses were larger and tended to peak at recording sites around apical and basal stimulating electrodes. This suggested a limited spread of excitation. Campbell et al. (2015) recorded from CI patients who retained audiometric thresholds between 75 and 90 dB HL at 500 Hz in their implanted ear. In response to acoustical stimulation they obtained eCAPs including CM and SP responses. The eCAP thresholds were similar to the audiometric thresholds. Dalbert et al. (2015b) used eCAPs to follow the post-surgery changes in hearing in CI patients, which were largely due to middle ear effusion, resulting from the surgery and disappeared over time.

From their modeling studies, Briaire and Frijns (2005) noted that the calculated eCAPs based on the theoretical unit response did not match the measured human eCAP obtained using neural response telemetry (Frijns et al., 2002). Briaire and Frijns (2005) found the potential solution to the discrepancy from a study by Miller et al. (2004) that indicated that two APs are present, and that the initial positive peak, when present, in the eCAP originates from antidromic APs originating from a relatively central site on the nerve fiber, likely close the ganglion cell body, of AP initiation. Thus, the dendrite may be responsible for the generation of the P_0_ peak. Note that in acoustic stimulation the site of initial spike excitation is likely the proximal dendrite (Hossain et al., 2005).

The study by Miller et al. (2004) indicated that the state of neural degeneration of the fibers has a big influence on the presence of the P_0_ peak in the unit response, as also implied by Rattay et al. (2001). Briaire and Frijns (2006) used this to show that a large P_0_ peak in the eCAP occurs before the N_1_P_1_ complex when the fibers are not degenerated. They suggested that the absence of this peak might be used as an indicator for degeneration of the proximal dendrite. Westen et al. (2011) evaluated the use of the unit response as a unitary response in a convolution integral to predict the eCAP and found evidence for changes in the unit response with stimulus level. This suggested that the unit responses for different electrodes are not independent, likely caused by strong synchronization across fibers at high stimulus levels. Therefore the eCAP cannot be predicted from the unit responses, and consequently, the inverse problem assessing the patency of the ANFs on basis of the eCAP is not unambiguous.

Recently, Strahl et al. (2016) used a deconvolution model to estimate the nerve firing probability based on a biphasic unit response and the eCAP, both in guinea pigs and human implantees. They found that the estimated nerve firing probability was bimodal and could be parameterized by two Gaussian distributions with an average latency difference of 0.4 ms. The ratio of the scaling factors of the late and early component increased with neural degeneration in the guinea pig. The two-component firing probability was attributed to either latency differences in the population of nerve fibers resulting from late firing due to excitation of the proximal dendrite, compared to direct, central to the cell body, activation of the ANFs. They suggested that the deconvolution of the eCAP could be used to reveal these two separate firing components in the auditory nerve, which may elucidate degeneration of the proximal dendrite.

Intraoperative recording from the round window or from the promontory during cochlear implant surgery has also been reported about in a recent series of articles (Mandalà et al., 2012; Calloway et al., 2014; McClellan et al., 2014; Dalbert et al., 2015a; Formeister et al., 2015; Adunka et al., 2016). I will not dwell on this ECochG use as it will be part of another set of articles in this Special Topic.

### Auditory Neuropathy

The diagnosis of “auditory neuropathy” usually does not require more than the presence of a superficial phenomenology consisting of recordable OAEs and absent or very poorly defined ABRs. Patients with auditory neuropathy also may have mild to moderate hearing loss and more severe speech perception deficits than expected based on the audiogram. However, there is quite a bit more differentiation with respect to underlying genetic and peripheral hearing mechanisms. This has lead among others to use of a new term “synaptopathy”, which puts one of the mechanism in the IHC ribbon synapses (Khimich et al., 2005; Kujawa and Liberman, 2009; Moser et al., 2013). It should be noted that acquired synaptopathy (Kujawa and Liberman, 2009) is completely different from that resulting from the *OTOF* mutation. Acquired synaptopathy resulting from a TTS following noise exposure, shows normal otoacoustic emission, normal ABR thresholds and waveforms and putatively a reduction in wave I amplitude at high stimulus levels. It is obvious that in such cases the CM, SP and CAP will all be normal, with a putative reduction in CAP amplitude at high stimulus levels, although this has been disputed (Bourien et al., 2014).

Another umbrella term is “dys-synchrony”, which can describe anything from the non-synchronous transmitter substance release from the ribbon synapses, resulting in onset desynchrony in the ANF firings, to changes in the peripheral dendrite of the spiral ganglion slowing down of APs along the ANFs (Rance and Starr, 2015) which also results in a large spread of spike latencies and hence poorly shaped ABRs. Ears affected by auditory neuropathy show a large CM riding on a large positive potential, presumably and SP^+^ (Gibson and Sanli, 2007).

Harrison (1998) found that scattered IHC loss, resulting from carboplatin administration in chinchillas resulted in normal oto-acoustic emissions, and CM whereas ABR thresholds were significantly elevated. He suggested that this type of damage could also result from longterm cochlear hypoxia and be a likely candidate for certain types of auditory neuropathy in humans.

Genes underlying two common forms of auditory neuropathy are *OTOF* resulting in synaptopathy and *OPA1* resulting in neuropathy of the spiral ganglion dendrites. Because IHC exocytosis was almost completely abolished in an *otoferlin* knock-out mouse model, *otoferlin* should have a role in a late step of exocytosis from the ribbon synapses. *Otoferlin* appears to mediate the replenishment of the ready releasable vesicle pool, and plays a role in the vesicle recruitment to the active zone membrane (Wichmann, 2015).

Huang et al. (2012) recorded the cochlear potentials CM, SP and CAPs by ECochG before cochlear implantation in patients diagnosed with familial optic atrophy which suggested an auditory neuropathy. Genetic analysis identified a R445H mutation in the *OPA1* gene. Audiological studies showed preserved DPOAEs and absent or abnormally delayed ABRs. TT ECochG showed prolonged low amplitude negative potentials without auditory nerve CAPs. After cochlear implantation, hearing thresholds, speech perception and synchronous activity in auditory brainstem pathways were restored. This suggests that deafness accompanying this OPA1 mutation is due to altered function of the dendritic portions of the spiral ganglion.

Santarelli et al. (2009) recorded abnormal click-evoked cochlear potentials with TT ECochG from four children with *OTOF* mutations to evaluate the physiological effects resulting from abnormal neurotransmitter release by IHCs. The children were profoundly deaf with absent ABRs and preserved otoacoustic emissions consistent with auditory neuropathy. Cochlear potentials evoked by clicks from 60 dB p.e. SPL to 120 dB p.e. SPL were compared to recordings obtained from 16 normally hearing children. The CM showed normal amplitudes from all but one ear, consistent with the preserved DPOAEs. After canceling the CM, the remaining cochlear potentials were of negative polarity with reduced amplitude and prolonged duration compared to controls. These cochlear potentials were recorded as low as 50–90 dB below behavioral thresholds in contrast to the close correlation in normal hearing controls between cochlear potentials and behavioral threshold (see Figure 4). SPs were identified in five out of eight ears with normal latency whereas CAPs were either absent or of low amplitude. Stimulation at high rates reduced amplitude and duration of the prolonged potentials, consistent with their neural generation site and not comprising SP^−^s. The remaining low-amplitude prolonged negative potentials are consistent with sustained exocytosis and decreased phasic neurotransmitter release (Khimich et al., 2005) resulting in abnormal dendritic activation and impairment of auditory nerve firing. This study suggests that mechano-electrical transduction and cochlear amplification are normal in patients with *OTOF* mutations.

**Figure 15:**
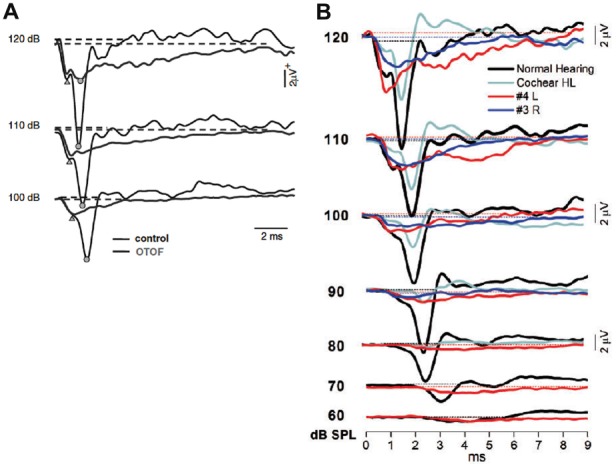
**(A)** Comparison between the SP–CAP potentials recorded from one subject with *OTOF* mutations and one control. The curves for the *OTOF* subject are superimposed on the recordings obtained from one control at intensities up to 120 dB p.e. SPL to highlight the similarities of the SP component between controls and patients with *OTOF* mutations. Open circles and triangles refer to the CAP and SP peaks, respectively. From Santarelli et al. (2009). **(B)** ECochG waveforms obtained after CM cancellation from two representative *OPA1* patients are superimposed on the corresponding responses recorded from one normal hearing control and from one hearing-impaired child with cochlear hearing loss (Cochlear HL) at decreasing stimulus intensity. From Santarelli et al. (2015).

Santarelli et al. (2013) then compared acoustically- and electrically-evoked potentials of the auditory nerve in patients with postsynaptic or presynaptic auditory neuropathy with underlying mutations in the *OPA1* or *OTOF* gene, respectively. Among non-isolated auditory neuropathy disorders, mutations in the *OPA1* gene are believed to cause disruption of auditory nerve discharge by affecting the unmyelinated portions of human ANFs. TT ECochG was used to record click-evoked responses from two adult patients carrying the R445H *OPA1* mutation, and from five children with mutations in the *OTOF* gene. The CM amplitude was normal in all subjects. Prolonged negative responses were recorded as low as 50–90 dB below behavioral threshold in subjects with *OTOF* mutations (Figure 15A) whereas in the *OPA1* disorder the prolonged potentials were correlated with hearing threshold (Figure 15B). A CAP was superimposed on the prolonged activity at high stimulation intensity in two children with mutations in the *OTOF* gene while CAPs were absent in the *OPA1* disorder. Electrically-evoked eCAPs (see “Cochlear Implants” Section) could be recorded from subjects with *OTOF* mutations but not from *OPA1* mutations following cochlear implantation (Santarelli et al., 2015).

Santarelli et al. (2015) further characterized the hearing dysfunction in *OPA1*- linked disorders. Nine of 11 patients carrying *OPA1* mutations inducing haplo-insufficiency had normal hearing function. Eight patients carrying *OPA1* missense variants underwent cochlear implantation. The use of cochlear implant improved speech perception in all but one patient. ABRs were recorded in response to electrical stimulation in five of six subjects, whereas no eCAP was evoked from the auditory nerve through the cochlear implant. These findings corroborate that the impaired mechanism in patients carrying *OPA1* missense variants is desynchronized ANF firings resulting from neural degeneration affecting the terminal dendrites (Santarelli et al., 2015).

## Summary

Ruben (1967)’s three important topics in ECochG were: (1) The correlation of physiological and psychoacoustic properties. (2) The investigation of certain diseases. (3) The objective diagnosis of individual cases of deafness. After 50 years we can make up the balance of the outcome of these three points.

Point one includes objective audiometry, which is quite accurate but is largely superseded by the non-invasive ABR. ECochG may remain the method of choice when objective hearing test have to be done under anesthesia. One may also say that intra-operative monitoring falls in this category. This likely becomes an important topic in relation to cochlear implantation. Several important differences between human and animal electrophysiology were found in some temporal response properties, such as adaptation and forward masking. Here the human data showed much larger time constants than those in common experimental animals. However, in these cases the human psychoacoustic data did not show any difference from the animal electrophysiological data. This requires further investigation. In addition the purported relation between oto-acoustic emission and CM needs more detailed study. Correlating the recorded eCAPs with a CI with applicable psychoacoustics needs to be further explored.

Point two, the investigation of certain diseases has been largely focused on Ménière’s disease, and has shown that for hearing losses up to 50 dB the OHC are not affected—normal SP and CM—and do not cause the fluctuating hearing loss. More promise hold the recent investigations of various genetic forms of auditory neuropathy, where ECochG powerfully illustrates the effects of the pre- and post-synaptic mechanisms on the temporal aspects of auditory nerve activity.

Point three, the objective diagnosis of individual cause of deafness, has focused primarily on vestibular schwannoma and Ménière’s disease, which show comparable broad and long lasting SP–CAP waveforms. ECochG highlighted the different underlying causes as relatively—compared to the CAP—large SP (Ménière’s disease) and monophasic unit contributions (vestibular schwannomas), respectively. However, the specificity and sensitivity of ECochG in these disorders has so far precluded reliable diagnosis in individual cases.

Point four, given the ambiguities of distinguishing SP^−^ from a desynchronized CAP in auditory neuropathy, and the interpretation of CM as a purely presynaptic potential, it is obvious that further basic research is needed into the limits of applicability of these traditionally considered “isolatable responses” in ECochG.

## Author Contributions

The author confirms being the sole contributor of this work and approved it for publication.

## Conflict of Interest Statement

The author declares that the research was conducted in the absence of any commercial or financial relationships that could be construed as a potential conflict of interest.
